# Nutritional and Inflammatory Markers Associated with Complete Response to Near-Infrared Photoimmunotherapy in Recurrent Head and Neck Squamous Cell Carcinoma

**DOI:** 10.3390/cancers18061022

**Published:** 2026-03-21

**Authors:** Hitoshi Hirakawa, Taro Ikegami, Hidetoshi Kinjyo, Shinya Agena, Hironori Nakayoshi, Takahiro Miyahira, Shunsuke Kondo, Norimoto Kise, Yuki Kayo, Hiroyuki Maeda, Mikio Suzuki

**Affiliations:** Department of Otorhinolaryngology, Head and Neck Surgery, Graduate School of Medicine, University of the Ryukyus, 1076 Kiyuna, Ginowan 901-2720, Okinawa, Japan; ikegami@cs.u-ryukyu.ac.jp (T.I.); h111853@cs.u-ryukyu.ac.jp (H.K.); h052482@cs.u-ryukyu.ac.jp (S.A.); nakayoshi_fwl2@cs.u-ryukyu.ac.jp (H.N.); miyahira_fzva@cs.u-ryukyu.ac.jp (T.M.); h082544@cs.u-ryukyu.ac.jp (S.K.); h115508@cs.u-ryukyu.ac.jp (N.K.); kayo_fls4@cs.u-ryukyu.ac.jp (Y.K.); hmaeida@cs.u-ryukyu.ac.jp (H.M.); suzuki@cs.u-ryukyu.ac.jp (M.S.)

**Keywords:** near-infrared photoimmunotherapy, recurrence, head and neck cancer, biomarker, nutritional marker, inflammatory marker, peripheral blood sample

## Abstract

**Background/Objectives**: Near-infrared photoimmunotherapy (NIR-PIT) provides tumor-selective cytotoxicity with minimal collateral tissue damage and has emerged as a novel treatment option for recurrent head and neck squamous cell carcinoma (HNSCC). However, biomarkers that predict treatment response to NIR-PIT remain poorly defined. Therefore, this study aimed to exploratorily determine whether baseline nutritional and inflammatory composite biomarkers are associated with complete response to NIR-PIT in patients with recurrent HNSCC. **Methods**: Fifteen non-surgical candidates with recurrent HNSCC underwent NIR-PIT between January 2022 and December 2025. Baseline composite nutritional indices and inflammatory markers, including the systemic inflammation response index (SIRI), were assessed before and 4–8 weeks post-treatment. Tumor response was evaluated according to RECIST version 1.1. Exploratory comparisons between complete response (CR) and non-CR groups were performed using Wilcoxon rank-sum tests with effect size estimation. **Results**: Five of 15 patients achieved CR (33.3%). Baseline SIRI was significantly lower in the CR group than in the non-CR group (median 70.7 vs. 120.2; *p* = 0.03), with a large effect size (r = 0.55). In contrast, baseline composite nutritional indices and other inflammatory markers showed no significant association with treatment response. Nutritional status remained stable after NIR-PIT, as reflected by preserved nutritional index values. SIRI tended to increase post-treatment in patients who achieved CR. **Conclusions**: NIR-PIT achieved encouraging local tumor responses in recurrent HNSCC while preserving early nutritional status. Baseline SIRI may represent a potential inflammation-based correlate of CR, reflecting the balance between systemic inflammation and host immune status, and warrants validation in larger prospective cohorts.

## Simple Summary

Recurrent head and neck cancer remains challenging to manage, particularly in patients for whom salvage surgery or re-irradiation is not suitable. Near-infrared photoimmunotherapy (NIR-PIT) has recently been introduced as a tumor-selective treatment option; it may stimulate the immune system and spare normal tissues. However, which patients benefit the most remains unclear, and little is known about how this treatment affects nutritional status, which is important for quality of life and treatment continuation. This study analyzed 15 patients treated with near-infrared photoimmunotherapy. Patients with lower systemic inflammation before treatment were more likely to achieve a complete response. Conversely, nutritional measurements remained stable after treatment, suggesting that near-infrared photoimmunotherapy did not worsen nutritional condition in the short term. These findings highlight inflammation as a potential predictive factor for treatment benefit and support the nutritional safety of near-infrared photoimmunotherapy.

## 1. Introduction

Head and neck squamous cell carcinoma (HNSCC) is the sixth most common cancer worldwide, with more than 800,000 new cases and approximately 450,000 deaths reported annually [1]. Despite advances in surgery, radiotherapy, and systemic therapy, recurrent disease continues to pose a major clinical challenge, particularly for patients not suited for salvage surgery or re-irradiation. In this population, survival outcomes remain poor, and maintaining quality of life and functional status while achieving tumor control is a critical therapeutic goal [2,3,4].

The use of immune checkpoint inhibitors (ICIs) has expanded treatment options and improved survival outcomes in recurrent and metastatic HNSCC; however, overall response rates remain modest [5]. Therefore, additional strategies that enhance tumor control while minimizing systemic toxicity are needed. Near-infrared photoimmunotherapy (NIR-PIT)—that selectively destroys epidermal growth factor receptor (EGFR)-expressing tumor cells through photoactivation of an antibody–photosensitizer conjugate—has emerged as a promising treatment modality [6]. Notably, NIR-PIT induces immunogenic cell death, promotes antigen release, and may synergize with immune modulation, including ICI therapy [7,8]. In Japan, NIR-PIT is clinically reimbursed on a per-lesion basis and can be administered up to four times per target lesion, enabling repeated local therapy while preserving surrounding tissues.

Host nutritional and inflammatory status are important determinants of cancer outcomes. Malnutrition and systemic inflammation impair immune competence, reduce treatment tolerance, and are associated with poorer prognosis across multiple tumor types [9,10]. Importantly, composite indices derived from routine peripheral blood tests—such as the Geriatric Nutritional Risk Index (GNRI) [11], Controlling Nutritional Status (CONUT) score, Prognostic Nutritional Index (PNI) [12], Systemic Immune-Inflammation Index (SII), and Systemic Inflammation Response Index (SIRI) [13]—offer practical and cost-effective approaches for evaluating the balance between nutritional and inflammatory status and have demonstrated prognostic relevance in lung, gastrointestinal, and head and neck cancers.

However, whether these markers can predict treatment response to NIR-PIT in recurrent HNSCC remains unclear. Because the clinical response to NIR-PIT varies across patients, identifying reliable biomarkers that can predict treatment response before therapy is clinically important for appropriate patient selection and treatment optimization.

Although NIR-PIT can be administered repeatedly, its treatment tolerance has not been well characterized. In particular, the preservation of nutritional status and changes in systemic inflammation post-therapy remain insufficiently understood [9]. The deterioration of nutritional status can limit tolerance to subsequent systemic therapies, ultimately compromising long-term treatment continuity.

Therefore, this retrospective study aimed to exploratorily evaluate nutritional and inflammatory biomarkers primarily as predictors of treatment response to NIR-PIT in non-surgical candidates with recurrent HNSCC. In addition, short-term changes in nutritional and inflammatory parameters after treatment were analyzed as secondary endpoints.

## 2. Materials and Methods

### 2.1. Study Design and Ethics Approval

This retrospective observational study included consecutive patients with recurrent HNSCC who underwent near-infrared photoimmunotherapy (NIR-PIT) at a single tertiary referral center between January 2022 and December 2025. Patients were treated under off-guideline clinical decision-making because standard curative options were not feasible. The study protocol was approved by the Institutional Review Board of the University of the Ryukyus (approval no. 1860) and conducted in accordance with the Declaration of Helsinki. Owing to the study’s retrospective nature, the requirement for written informed consent was waived, and an opt-out system was implemented via the institutional website.

### 2.2. Patient Eligibility and Baseline Assessment

Eligible patients were non-surgical candidates with locoregionally recurrent HNSCC who had previously received radiotherapy and showed no evidence of distant metastasis at the time of receiving NIR-PIT. Patients with tumors invading or abutting the carotid artery were excluded because of the potential risk of catastrophic hemorrhage associated with rapid tumor necrosis. All patients had an Eastern Cooperative Oncology Group performance status of 0 or 1 and adequate organ function confirmed within 21 days before treatment initiation.

### 2.3. Multidisciplinary Decision-Making and Clinical Data Collection

Resectability and treatment eligibility were determined through a multidisciplinary discussion involving head and neck surgeons, radiation oncologists, reconstructive surgeons, medical oncologists, and oral and maxillofacial surgeons. Anatomical constraints, functional preservation, comorbid conditions, and patient preference were used to make decisions. Physical examination, endoscopy, contrast-enhanced computed tomography (CT), magnetic resonance imaging (MRI), and positron emission tomography–CT were used for baseline evaluation, as appropriate. Tumors were staged according to the Union for International Cancer Control TNM Classification, 8th edition.

### 2.4. NIR-PIT Procedure and Treatment Sequencing

NIR-PIT was performed following standard clinical protocols. Patients received an intravenous infusion of cetuximab sarotalocan sodium (640 mg/m^2^) approximately 20–28 h before near-infrared laser irradiation. Laser irradiation was delivered using cylindrical or frontal diffusers selected based on tumor size, depth, and location.

In a subset of patients, pembrolizumab was administered as prior ICI therapy more than one month before NIR-PIT at the discretion of the treating physicians. Baseline blood samples were obtained before NIR-PIT and at least one month after the last pembrolizumab administration.

### 2.5. Assessment of Response and Safety

Tumor response was assessed using imaging studies and endoscopic evaluation according to RECIST version 1.1 [14]. Disease progression was determined radiologically or clinically during follow-up. Adverse events were recorded and graded using the National Cancer Institute Common Terminology Criteria for Adverse Events (CTCAE), version 4.0.

### 2.6. Nutritional and Inflammatory Biomarkers

Peripheral blood samples were collected at baseline and after NIR-PIT. The post-treatment sampling date (typically within 4–8 weeks) was determined in the outpatient setting based on the treating physician’s judgment and corresponded to the time point when local acute inflammatory reactions at the NIR-PIT–treated site (e.g., necrosis, secondary infection, or treatment-related wound inflammation) had resolved. This approach was taken to minimize the confounding influence of acute local inflammatory changes and isolate the systemic nutritional and inflammatory responses attributable to NIR-PIT itself.

Nutritional markers—GNRI [15], CONUT score [16], PNI [17], SII [18], and SIRI [13]—were calculated using established formulas derived from routine laboratory parameters. Changes in biomarker values before and after treatment were analyzed to assess nutritional and inflammatory stability following NIR-PIT.

### 2.7. Statistical Analysis

Continuous variables were compared using the Mann–Whitney U test, and categorical variables were analyzed using Fisher’s exact test. Effect sizes were calculated for nonparametric comparisons. Given the study’s exploratory nature and the limited sample size, no adjustments for multiple comparisons were applied, and interpretation emphasized effect sizes in addition to *p*-values. Effect sizes were calculated using the rank-based correlation coefficient r [19].

## 3. Results

### 3.1. Patient Characteristics and Treatment Overview

Fifteen non-surgical candidates with recurrent HNSCC were included in this analysis. All patients had previously undergone radiotherapy and had no evidence of distant metastasis at the time of NIR-PIT. Baseline demographic and clinical characteristics are summarized in Table 1. The median age was 62 years (range, 48–83), and the majority of patients were male. Tumor locations were predominantly the oropharynx and oral cavity, but also included the nasopharynx, maxillary sinus, and cervical lymph nodes.

Five of 15 patients had received prior pembrolizumab-based chemotherapy.

### 3.2. Treatment Details

NIR-PIT was administered for 25 treatment cycles to 15 patients.

**Figure 1:**
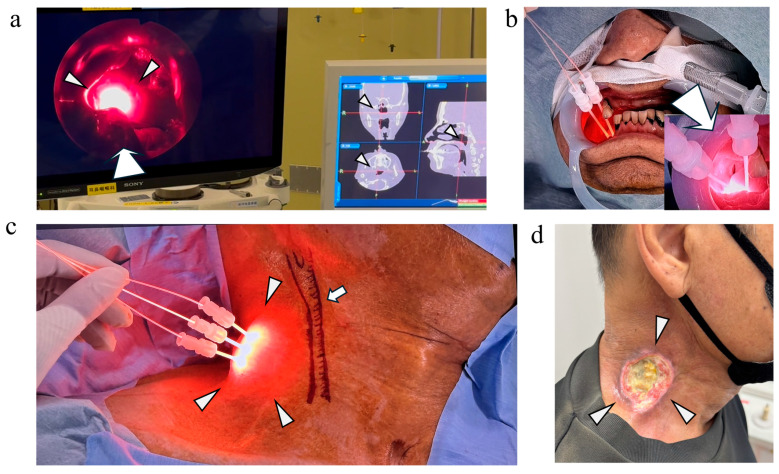
Representative technical approaches for NIR-PIT delivery. (**a**) In Case 7, the near-infrared laser illumination of a nasopharyngeal carcinoma was performed under endoscopic visualization using a sinonasal endoscope and a navigation system, with a flexible device equipped with frontal diffusers. Arrowheads indicate the nasopharyngeal carcinoma, and the arrow denotes the flexible frontal-diffuser device. (**b**) In Case 8, the transoral insertion of a cylindrical diffuser was performed for a lower gingival squamous cell carcinoma in a patient with severe trismus caused by tumor progression, which resulted in complete response (CR). Endoscopic assistance (arrow) was required to secure adequate visualization and safe access because of the markedly limited mouth opening. (**c**,**d**) In Case 14, a percutaneous approach was used for recurrent deep cervical lymph node metastasis (arrowhead) arising from oropharyngeal cancer after neck dissection and postoperative radiotherapy, representing a particularly challenging clinical scenario. As shown in panel (**c**), the position and course of the common carotid artery (arrow) were visualized to maintain continuous spatial awareness and to avoid needle advancement toward it. The puncture plan was carefully designed by anticipating the potential extent of post-treatment tumor necrosis to prevent inadvertent involvement of the carotid artery (**d**). Using this strategy, the tumor target lesion (arrowhead) was safely treated without carotid artery rupture, resulting in a partial response.

Representative technical approaches for NIR-PIT delivery are shown in Figure 1.

In Case 7, the near-infrared laser illumination of a nasopharyngeal carcinoma was performed under endoscopic visualization using a sinonasal endoscope and a navigation system, with a flexible device equipped with frontal diffusers. (Figure 1a). In Case 8, a cylindrical diffuser was inserted transorally to ensure safe and accurate placement for a lower gingival tumor, resulting in a complete response (CR). (Figure 1b). In Case 14, ultrasound-guided percutaneous insertion was used for deep cervical nodal disease associated with oropharyngeal cancer, resulting in partial response (PR) (Figure 1c,d). In some cases, NIR-PIT was repeated for the same lesion, with up to three sessions administered per lesion.

### 3.3. Tumor Response and Clinical Outcomes

Tumor response was evaluated according to RECIST version 1.1, and best overall response (BOR) was recorded. Among the 15 patients, CR was achieved in 5 patients (33.3%), PR in 5 patients, stable disease (SD) in 2 patients, and PD in 3 patients. The overall response rate (ORR; CR + PR) was 66.6% (Table 2).

### 3.4. Stratification by Response

For subsequent biomarker analyses, patients were stratified into a CR group (*n* = 5) and a non-CR group (*n* = 10). Associations between clinical characteristics, treatment-related factors, and CR to NIR-PIT are summarized in Table 3. No baseline demographic factors, including age, smoking status, alcohol consumption, tumor subsite, clinical stage, performance status, mGPS and aCCI, were significantly associated with achieving CR. Similarly, treatment-related variables, including the number of NIR-PIT treatment cycles, device type, and occurrence of adverse events, showed no significant correlation with CR.

The non-CR rate was lower in patients with prior ICI exposure than in those without prior ICI exposure (20% vs. 80%); however, this difference did not reach statistical significance (odds ratio 6.0, 95% CI 0.56–63.9; *p* = 0.26). Overall, these findings indicate that conventional clinical and treatment-related factors were not predictive of CR in this cohort.

### 3.5. Baseline Nutritional Biomarkers

Baseline nutritional indices, including the GNRI, CONUT score, and PNI, showed no statistically significant differences between the CR and non-CR groups (Table 2). Effect size estimates for GNRI and CONUT were small, indicating limited baseline discrimination between responders and non-responders.

### 3.6. Inflammatory Biomarkers and Response

**Figure 2:**
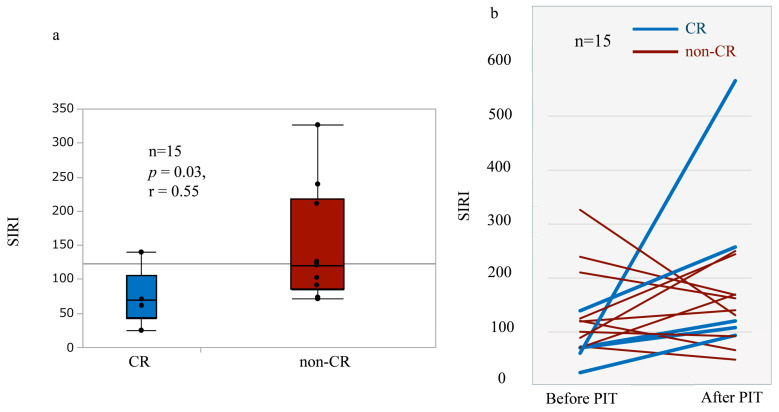
Baseline SIRI and longitudinal changes in SIRI according to treatment response. (**a**) Comparison of baseline Systemic Inflammation Response Index (SIRI) values between patients who achieved complete response (CR) and those with non-complete response (non-CR). Baseline SIRI was significantly lower in the CR group (*p* = 0.03), with a large effect size (r = 0.55). (**b**) Individual trajectories of SIRI values before and after near-infrared photoimmunotherapy (NIR-PIT), stratified by response category. All patients who achieved CR showed an increase in SIRI following treatment, whereas patients with non-complete response (non-CR) exhibited heterogeneous post-treatment changes, including both increases and decreases.

In this exploratory analysis, pre-treatment SIRI was significantly lower in patients who achieved CR compared with the non-CR group (median 70.7 vs. 120.2; *p* = 0.03), with a large effect size (r = 0.55) (Table 4, Figure 2a). SII demonstrated a similar but non-significant directional trend.

Longitudinally, SIRI increased after NIR-PIT in all patients who achieved CR, whereas those in the non-CR group exhibited heterogeneous post-treatment trajectories, including both increases and decreases. In this exploratory analysis, these findings suggest that both baseline inflammatory burden and its dynamic modulation during therapy may be associated with treatment response (Figure 2b).

Post-treatment blood samples were collected after resolution of acute local inflammatory reactions at the treated sites. SIRI, Systemic Inflammation Response Index; CR, complete response; non-CR, non-complete response; NIR-PIT, near-infrared photoimmunotherapy.

### 3.7. Changes in Nutritional and Inflammatory Status Following NIR-PIT

**Figure 3:**
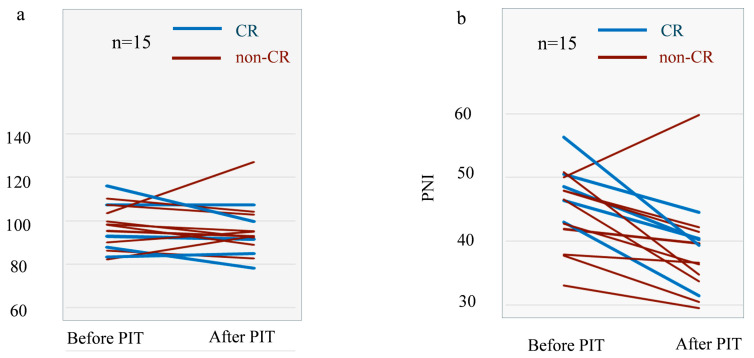
Stability of nutritional status before and after NIR-PIT. (**a**) Individual trajectories of the geriatric nutritional risk index (GNRI) evaluated before and 4–8 weeks after near-infrared photoimmunotherapy (NIR-PIT). GNRI values showed no evidence of nutritional deterioration at the group level, with a median change of −3.22 and a median percentage change of −2.9%, indicating preserved nutritional status during the early post-treatment period. (**b**) Individual trajectories of the prognostic nutritional index (PNI) over the same period. Although modest numerical declines were observed (median change −6.31; median percentage change −13.5%), no patient crossed clinically relevant thresholds for malnutrition or experienced clinically meaningful functional decline, supporting overall nutritional preservation following NIR-PIT. GNRI, Geriatric Nutritional Risk Index; PNI, Prognostic Nutritional Index; NIR-PIT, near-infrared photoimmunotherapy.

Longitudinal changes in nutritional indices were assessed between baseline and after treatment to evaluate whether NIR-PIT adversely affected nutritional status. Individual trajectories of GNRI are shown in Figure 3a. Overall, GNRI values appeared relatively stable after NIR-PIT, with a median change of −3.22 and a median percentage change of −2.9%, and no statistically significant decline was observed (*p* = 0.46). Similar stability was observed for PNI, with a median change of −6.31 and a median percentage change of −13.5% (Figure 3b). Within the early post-treatment period assessed in this exploratory study, no patient experienced clinically meaningful deterioration in nutritional status.

**Figure 4:**
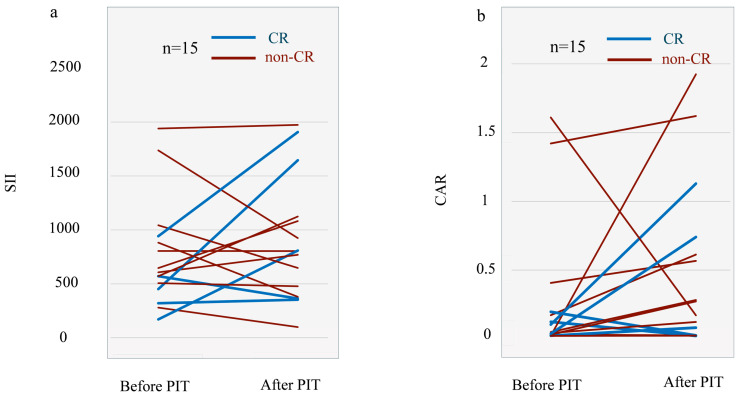
Changes in systemic inflammatory indices before and after NIR-PIT. (**a**) Systemic Immune-Inflammation Index (SII) values assessed before and after near-infrared photoimmunotherapy (NIR-PIT) showed heterogeneous individual changes without consistent worsening. The median change in SII was +32.6, and the median percentage change was +1.7%, indicating only mild fluctuations in the early post-treatment period. (**b**) C-reactive protein-to-albumin ratio (CAR) values demonstrated similarly modest changes, with a median change of +0.16 and no significant difference observed between pre- and post-treatment measurements (*p* = 0.20). These findings suggest that NIR-PIT does not induce uniform deterioration in systemic inflammatory status during the early post-treatment phase. SII, Systemic Immune-Inflammation Index; CAR, C-reactive protein-to-albumin ratio; NIR-PIT, near-infrared photoimmunotherapy.

Individual trajectories of SII are shown in Figure 4a. Overall, no uniform worsening of systemic inflammatory markers was observed after treatment. SII exhibited heterogeneous individual changes, with a median change of +32.6 and a median percentage change of +1.7%, indicating mild increases in some patients and stable or decreased values in others. At the group level, no significant difference was observed between pre- and post-treatment SII values.

Similarly, changes in CAR were modest, with a median change of +0.16, and no significant difference was detected between pre- and post-treatment C-reactive protein-to-albumin ratio (CAR) values, *p* = 0.20, Figure 4b).

### 3.8. Overall Survival and Safety Profile

**Figure 5:**
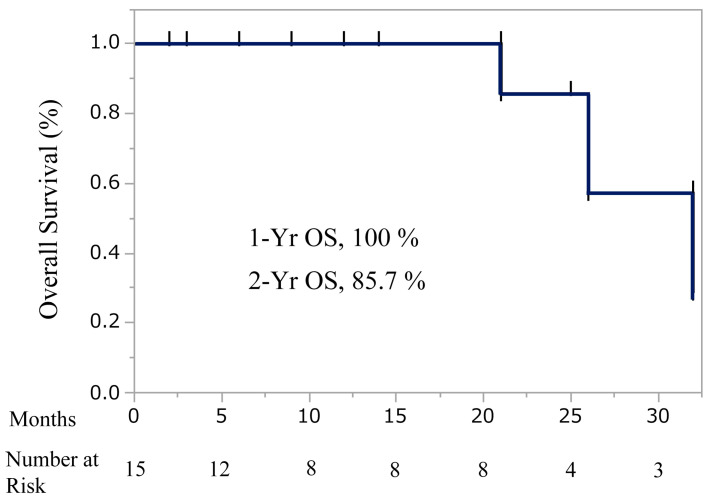
Kaplan–Meier estimation of cumulative overall survival.

**Figure 6:**
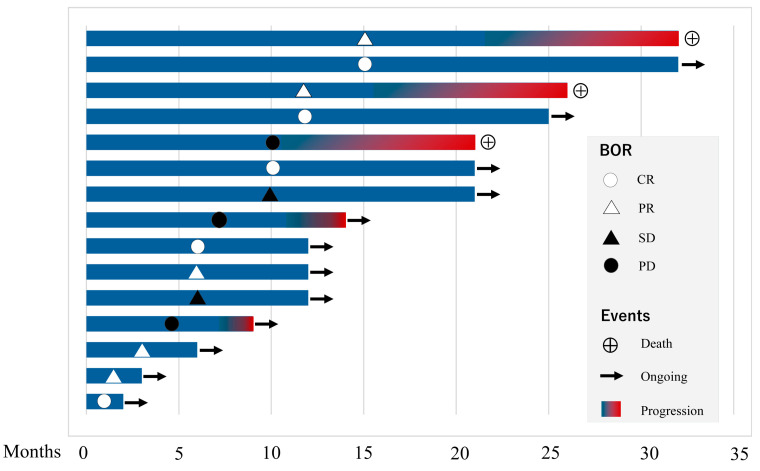
Swimmer’s plot illustrating individual treatment courses and clinical outcomes following near-infrared photoimmunotherapy (NIR-PIT).

Based on Kaplan–Meier estimates, the 1-year overall survival rate was 100%, and the 2-year overall survival rate was 85.7% (Figure 5). A swimmer’s plot additionally illustrated individual patient courses and treatment responses over time (Figure 6).

NIR-PIT was generally well tolerated. The most frequently observed adverse events were grade 1–2 local pain and edema at the treatment site. Grade 3 adverse events occurred in three patients and were primarily related to local inflammation and were managed conservatively. No treatment-related deaths or immune-related adverse events were observed during the study period.

Notably, adverse events were observed in all patients among cases 1–7, and all grade 3 events occurred within this early cohort. In contrast, among cases 8–15, adverse events were documented in four patients, all limited to grade 1 pain, and no adverse events of grade ≥ 2 were observed (Table 2).

At a median follow-up of 16.5 months (range, 2–32 months), the Kaplan–Meier analysis showed a 1-year overall survival (OS) rate of 100% and a 2-year OS rate of 85.7%.

Tick marks on the curve indicate censored observations, and the number of patients at risk at each time point is displayed below the x-axis.

Events and censoring were recorded based on the days from the initiation of NIR-PIT.

Each horizontal bar represents an individual patient and indicates the duration of follow-up after NIR-PIT. Best overall response (BOR) was assessed according to RECIST version 1.1 and is indicated by symbols: open circle, complete response (CR); open triangle, partial response (PR); filled triangle, stable disease (SD); filled circle, progressive disease (PD). Blue bars represent the treatment period, and the red gradient indicates the onset of disease progression. The circled cross symbol denotes death, while arrows indicate ongoing follow-up at the time of data cutoff. This swimmer’s plot highlights the heterogeneity of treatment responses and clinical trajectories among patients treated with NIR-PIT.

## 4. Discussion

In this study of recurrent HNSCC treated with NIR-PIT, we exploratorily found that baseline systemic inflammatory status—quantified by the SIRI—was associated with local tumor response. Patients with lower pre-treatment SIRI values were significantly more likely to achieve CR; baseline nutritional indices showed no association with tumor response.

### 4.1. SIRI as a Surrogate of Tumor–Immune Equilibrium

SIRI integrates neutrophil-mediated and monocyte-mediated protumor inflammation with lymphocyte-dependent antitumor immunity, thereby providing a composite inflammatory biomarker that captures systemic tumor–immune equilibrium more comprehensively than isolated single-parameter leukocyte indices, such as absolute neutrophil or lymphocyte counts [13,20,21]. Elevated baseline SIRI is associated with unfavorable long-term outcomes in various solid tumors, reflecting a chronic protumorigenic inflammatory milieu [22].

In this context, our finding that low baseline SIRI was associated with CR following NIR-PIT is biologically plausible and suggests that a systemic environment characterized by reduced myeloid-driven inflammation and preserved lymphocyte-mediated antitumor immunity is conducive to effective NIR-PIT–induced tumor eradication. These results suggest a potential clinical relevance of SIRI to a novel, locally applied, immunogenic treatment modality.

### 4.2. Post-Treatment SIRI Kinetics and Treatment Response

A novel observation in our cohort was the differential SIRI trajectories between response groups. Patients who achieved CR exhibited uniformly low baseline SIRI but demonstrated a post-treatment increase in SIRI following NIR-PIT. In contrast, non-CR patients had significantly higher baseline SIRI, and post-treatment changes were heterogeneous, with both increases and decreases observed.

Mechanistically, NIR-PIT induces rapid necrotic tumor cell death with release of damage-associated molecular patterns (DAMPs), tumor antigens, and chemotactic signals, thereby facilitating dendritic cell activation and subsequent T-cell priming [23,24]. In patients who achieved CR, the post-treatment rise in SIRI may reflect transient changes in innate immune cell dynamics during this process, which is consistent with an acute inflammatory response accompanying immunogenic cell death (ICD) [25,26]. Importantly, this increase should not be interpreted as detrimental inflammation; rather, it may represent treatment-related immune activation that is distinct from chronic protumor inflammation. Although NIR-PIT is a locally applied therapy, its mechanism involves immunogenic tumor cell death with potential systemic immune consequences. This provides a biological rationale for why baseline systemic inflammatory balance, as reflected by SIRI, and its early modulation after treatment, may be associated with local tumor response. However, these observations should be interpreted as exploratory, and the present analysis was limited to short-term post-treatment observation. It remains unclear whether the observed SIRI elevation is transient and normalized subsequently or whether it may evolve into sustained inflammation. Longer-term longitudinal studies will be required to clarify the clinical significance of post-treatment SIRI dynamics.

### 4.3. Nutritional Indices and Tumor Response

Contrary to substantial oncologic literature demonstrating that malnutrition adversely affects therapeutic efficacy [27,28], baseline GNRI, CONUT, and PNI did not clearly discriminate tumor response in our cohort. This finding may be explained by two factors: first, most patients had preserved performance status and organ function, resulting in limited variability in nutritional status; second, given the mechanism of NIR-PIT, short-term local tumor control may depend more on the baseline inflammatory–immune balance, as reflected by indices such as SIRI, than on nutritional reserve [29].

### 4.4. Clinical Course of Nutrition

GNRI, PNI, and CONUT remained stable 4–8 weeks after NIR-PIT, a notable finding, in contrast to chemoradiotherapy, which frequently induces mucositis, dysphagia, and weight loss [30].

NIR-PIT is characterized by a transient inflammatory response that resolves relatively early and, compared with definitive chemoradiotherapy or curative surgery, may exert less detrimental impact on nutritional status. Consistent with this notion, a previous study reported that NIR-PIT did not reduce quality of life in patients with unresectable head and neck cancer, a finding that may indirectly reflect preserved nutritional status.

Given that NIR-PIT can be administered repeatedly under the Japanese reimbursement system, nutritional preservation may support treatment continuity and facilitate sequential systemic therapies, including ICIs [8,31].

### 4.5. Safety Evolution and Procedural Learning Effects

From a safety standpoint, NIR-PIT was generally well tolerated; however, toxicity patterns appeared to evolve. Early cases (Case 1–7) experienced frequent adverse events (AEs), including all three grade 3 inflammatory events, which occurred exclusively within this group, whereas later cases (Case 8–15) exhibited milder toxicity limited to grade 1 pain without any grade ≥ 2 events. This pattern should be interpreted as a preliminary and anecdotal observation requiring further study rather than a definitive finding. Although the sample size precludes firm conclusions, several non–mutually exclusive explanations are possible, including differences in patient selection, tumor location, lesion complexity, and possible procedural learning effects. Importantly, NIR-PIT introduces a procedural paradigm fundamentally different from conventional surgery or radiotherapy; the technique requires surgeons to place a laser needle into the target lesion transorally or percutaneously, rather than performing mechanical excision or delivering external ionizing beams. Consequently, operator skill and familiarity with needle navigation, tissue planes, and perilesional anatomy may substantially influence safety and performance, suggesting a possible contribution of procedural learning and clinical implementation to real-world outcomes [31,32]. In addition, the risk of carotid blowout cannot be completely disregarded in this procedure, due either to inadvertent carotid puncture or postoperative inflammatory extension to perivascular tissue. Currently, no device enables real-time visualization of the needle tip with sufficient spatial precision, and even high-resolution neck ultrasonography may not provide complete safety assurance. Further technological advancements are therefore warranted to enhance procedural guidance and risk mitigation.

### 4.6. Interpretation and Considerations

The interpretation of these findings should be in light of some important limitations. First, the small sample size and single-institution retrospective design substantially limited generalizability and precluded definitive conclusions. In particular, the limited cohort size reduced statistical power and precluded multivariable analyses to adequately control for potential confounders. Prior immune checkpoint inhibitor (ICI) exposure may also be relevant when interpreting the association between baseline SIRI and response to NIR-PIT, as lower baseline SIRI and better treatment response may, at least in part, reflect the lasting immunomodulatory effects of prior ICI therapy rather than the effect of NIR-PIT alone. Although the interval between pembrolizumab and initiation of NIR-PIT exceeded 6 weeks in our cohort, suggesting that any direct residual effect may have been limited, persistent immunologic influences cannot be excluded. Second, biomarker analyses were restricted to peripheral blood and did not incorporate intratumoral immunologic correlates, limiting mechanistic insight into tumor–host interactions. Third, the short observation period focused on early tumor response rather than long-term oncologic outcomes.

Within these constraints, systemic inflammatory status and nutritional indices may capture distinct clinical dimensions, with the former potentially relating to tumor response and the latter to treatment tolerability. Accordingly, the present findings should be interpreted as exploratory. Prospective, multicenter validation integrating longitudinal biomarker assessment and intratumoral immune profiling will be essential to clarify biomarker utility and optimize the clinical application of NIR-PIT.

## 5. Conclusions

Our findings suggest that baseline systemic inflammation quantified by SIRI—reflecting an integrated balance between protumor inflammatory burden and host antitumor immune status—may represent a potentially clinically and biologically relevant correlate of local tumor control following NIR-PIT for recurrent HNSCC, whereas baseline nutritional indices were not predictive of response. Moreover, NIR-PIT achieved antitumor efficacy without marked early nutritional deterioration in this exploratory study.

## Figures and Tables

**Table 1:** Baseline patient and tumor characteristics.

Patient	Sex	Age	ECOG-PS	Tumor Location	rTNM ^a^	Target Tumor Size (mm)
1	Male	57	0	Oropharynx	T2N0M0	39 × 10 × 3
2	Male	59	1	Oropharynx	T3N0M0	47 × 42 × 36
3	Male	68	0	Oropharynx	T4N0M0	38 × 36 × 30
4	Male	64	0	Oral cavity	T2N0M0	31 × 26 × 20
5	Male	70	0	Oropharynx	T2N0M0	28 × 25 × 19
6	Male	66	1	Neck lymph node	T0N3bM0	40 × 38 × 36
7	Male	48	1	Nasopharynx	T3N0M0	42 × 32 × 32
8	Male	60	0	Oral cavity	T2N0M0	27 × 16 × 12
9	Male	62	0	Maxillary sinus	T2N0M0	21 × 17 × 11
10	Male	83	1	Oral cavity	T2N0M0	30 × 30 × 5
11	Male	62	0	Hypopharynx	T2N0M0	21 × 18 × 5
12	Female	80	1	Oropharynx	T4N0M0	46 × 41 × 20
13	Female	59	3	Oral cavity	T3N0M0	49 × 28 × 20
14	Male	55	1	Neck lymph node	T0N3bM0	37 × 28 × 26
15	Male	52	0	Nasopharynx	T1N0M0	14 × 7 × 5

^a^ According to the Union for International Cancer Control TNM classification. ECOG-PS: Eastern Cooperative Oncology Group Performance Status.

**Table 2:** Treatment details, best overall response, and adverse events of individual target lesions.

Patient	Target Tumor Lesion	Cycle	Device	BOR	AE	Sequential ICI	Final Clinical Outcome
1	Lateral wall of the oropharynx	3	CD + FD	PR	Aspiration pneumonia G3, Pain, G2	PIT → ICI	DOD
2	Lateral wall of the oropharynx	2	CD	CR	Pain, G2	PIT	NED
3	Base of tongue	3	CD	PR	Fistula, G3	PIT → ICI	DOD
4	Buccal mucosa	2	CD	PD	Trismus, G3, Fistula, G1, Pain, G1	PIT → ICI	DOD
5	Base of tongue	1	CD	CR	Fistula, G1, Facial edema, G2, Pain, G2	ICI → PIT	NED
6	Anterior neck lymph node	1	CD	CR		ICI → PIT	NED
7	Posterolateral wall of the nasopharynx	1	FD	SD		PIT → ICI	AWD
8	Floor of the mouth	2	CD	CR	Pain, G1	ICI → PIT	NED
9	Maxillary sinus	2	CD + FD	PD		PIT → ICI	AWD
10	Buccal mucosa	2	CD + FD	PR		PIT	AWD
11	Postcricoid region of the hypopharynx	2	FD	SD	Pain, G1	PIT → ICI	AWD
12	Base of tongue	1	CD	PD	Pain, G1	ICI → PIT	AWD
13	Lower gingiva	1	CD	PR	Pain, G1	PIT → ICI	AWD
14	Posterior neck lymph node	1	CD	PR		ICI → PIT	AWD
15	Posterolateral wall of the nasopharynx	1	FD	CR		PIT	NED

CD: cylindrical diffuser; FD: frontal diffuser; BOR: best overall response; CR: complete response; PR: partial response; SD: stable disease; PD, progressive disease; AE: adverse event; G: grade; ICI, immune checkpoint inhibitor; PIT, photoimmunotherapy; DOD, died of disease; NED, no evidence of disease; AWD, alive with disease.

**Table 3:** Clinical and treatment-related factors associated with complete response to NIR-PIT.

Characteristic	Category	Response to NIR-PIT (*n* = 15)		Odds Ratio (95% CI)	*p* Value
		CR (%)	Non-CR (%)		
Age		60 (52–70 years)	62 (48–83 years)	NA	0.90
Smoking	Present	3 (60)	6 (60)		
	Previous/No	2 (40)	4 (40)	1.0 (0.11–8.94)	1.00
Alcohol	Present	1 (20)	6 (60)		
	Previous/No	4 (80)	4 (40)	0.17 (0.01–2.09)	0.28
Tumor subsite	OPC/OC	3 (60)	6 (60)		
	Others	2 (40)	4 (40)	1.0 (0.11–8.95)	1.00
Clinical stage ^a^	I–II	3 (60)	5 (50)		
	III–IV	2 (40)	5 (50)	1.5 (0.17–13.22)	1.00
ECOG-PS	0	3 (60)	5 (50)		
	≥1	2 (40)	5 (50)	1.5 (0.17–13.22)	1.00
mGPS	A/C	3 (60)	7 (70)		
	B/D	2 (40)	3 (30)	0.64 (0.07–6.06)	1.00
aCCI	<4	3 (60)	2 (20)		
	>5	2 (40)	8 (80)	6.0 (0.56–63.98)	0.25
Previous ICI	Yes	3 (60)	2 (20)		
	No	2 (40)	8 (80)	6.0 (0.56–63.9)	0.26
Number of NIR–PIT cycles	1	3 (60)	4 (40)		
	≥2	2 (40)	6 (60)	2.25 (0.25–20.1)	0.61
NIR-PIT Device	CD	4 (80)	8 (80)		
	FD	1 (20)	2 (20)	1.0 (0.07–14.6)	1.00
AE	Yes	3 (60)	6 (60)		
	No	2 (40)	4 (40)	1.0 (0.11–8.95)	1.00

^a^ According to the Union for International Cancer Control TNM classification. NIR-PIT, near-infrared photoimmunotherapy; ECOG-PS, Eastern Cooperative Oncology Group performance status; mGPS, modified Glasgow Prognostic Score; ICI, immune checkpoint inhibitor; aCCI, age-adjusted Charlson Comorbidity Index; OPC, oropharyngeal cancer; OC, oral cavity cancer; NA, not applicable.

**Table 4:** Association of baseline nutritional and inflammatory indices with complete response.

Nutrition, Inflammatory Factor	Response to NIR-PIT (*n* = 15)			
	CR	Non-CR	Effect Size (r)	*p*-Value
BMI	18.6 (16.6–23.8)	21.9 (15.7–24.8)	0.1	0.67
Che	312 (175–436)	282 (207–421)	0.24	0.36
LDH	178 (151–332)	180 (126–377)	0.06	0.81
TG	104 (100–158)	93.5 (63–192)	0.21	0.43
TC	200 (179–254)	201 (127–244)	0.14	0.58
Alb	4.2 (3.7–5)	3.9 (2.8–4.6)	0.25	0.33
GNRI	92.9 (87.9–116.1)	98.2 (82.2–110.2)	0.06	0.80
PNI	46.4 (42.0–56.3)	47.3 (33.0–50.8)	0.14	0.54
CAR	0.11 (0.02–0.20)	0.05 (0.02–1.61)	0	1.00
SII	4509 (1701–9420)	7253 (2792–19,402)	0.4	0.13
SIRI	70.7 (24.7–139.5)	120.2 (71.1–326.3)	0.55	0.03
CONUT score				
≤2	4 (80)	5 (50)	NA	0.58
≥3	1 (20)	5 (50)		Odds ratio (95% CI): 0.25 (0.02–3.10)

NIR-PIT, near-infrared photoimmunotherapy; BMI, body mass index; Che, serum cholinesterase; LDH, lactate dehydrogenase; TG, triglycerides; TC, total cholesterol; Alb, serum albumin; GNRI, Geriatric Nutritional Risk Index; PNI, Prognostic Nutritional Index; CAR, C-reactive protein-to-albumin ratio; SII, Systemic Immune-Inflammation Index; SIRI, Systemic Inflammation Response Index; CONUT, Controlling Nutritional Status; CR, complete response; NA, not applicable.

## Data Availability

The original contributions presented in this study are included in the article. Further inquiries can be directed to the corresponding author.
